# Temporal trend in fetal mortality according to two death avoidability classifications

**DOI:** 10.1590/1980-220X-REEUSP-2024-0015en

**Published:** 2024-09-27

**Authors:** Rebeca Danielly Barros Xavier, Mirella Bezerra Rodrigues Vilela, Cristine Vieira do Bonfim, Conceição Maria Oliveira, Pedro Israel Cabral de Lira, Silvia Wanick Sarinho

**Affiliations:** 1 Secretaria de Estado da Saúde de Alagoas, Hospital da Mulher Dra. Nise da Silveira, Maceió, AL, Brazil.; 2 Universidade Federal de Pernambuco, Departamento de Fonoaudiologia, Recife, PE, Brazil.; 3 Fundação Joaquim Nabuco, Diretoria de Pesquisas Sociais. Recife, PE, Brazil.; 4 Secretaria de Saúde do Recife, Secretaria Executiva de Vigilância à Saúde. Recife, PE, Brazil.; 5 Universidade Federal de Pernambuco, Centro de Ciências da Saúde, Departamento de Nutrição. Recife, PE, Brazil.; 6 Universidade Federal de Pernambuco, Programa de Pós‐Graduação em Saúde da Criança e do Adolescente. Recife, PE, Brazil.

**Keywords:** Fetal Mortality, Causes of Death, Epidemiological monitoring, Time Series Studies., Mortalidad Fetal, Causas de Muerte, Monitoreo Epidemiológico, Estudios de series temporales

## Abstract

**Objective:**

To analyze the temporal trend of fetal mortality and its components, of avoidable and ill-defined causes according to two avoidability classifications in Recife, Pernambuco, 2010–2021.

**Method:**

Ecological study of temporal trends of fetal mortality in Recife, 2010–2021. The Brazilian List of Avoidable Causes of Death for fetal deaths (LBE-OF) and Brazilian List of Avoidable Causes of Death for children under five years of age (LBE < 5) were used. The *Joinpoint* regression model was applied to analyze the temporal trends.

**Results:**

Trends in fetal mortality and its components were stationary. The group of avoidable causes presented higher mortality rates in both classifications, with an increasing trend according to the LBE-OF (Annual Percentage Change-APC: 2,1; p = 0,018) and stationary according to the LBE < 5. There was a decreasing trend in mortality from ill-defined causes only according to the LBE-OF (APC: –12,3; p < 0,001).

**Conclusion:**

The results showed the stagnation of the temporal trend in fetal mortality, the avoidability of most deaths, and the potential of LBE-OF in monitoring the quality of information on the basic causes and avoidability of fetal deaths.

## RESUMEN

**Objetivo:**

Analizar la tendencia temporal de la mortalidad fetal y sus componentes, causas evitables y mal definidas según dos clasificaciones de evitabilidad en Recife, Pernambuco, 2010–2021.

**Metodología:**

Estudio ecológico de tendencias temporales de la mortalidad fetal en Recife, 2010–2021. Se utilizó la Lista Brasileña de Causas Evitables de Muerte para muertes fetales (LBE-OF) y la Lista Brasileña de Causas Evitables de Muerte para niños menores de cinco años (LBE < 5). Se aplicó el modelo de regresión *Joinpoint* para analizar las tendencias temporales.

**Resultados:**

Las tendencias en la mortalidad fetal y sus componentes se mantuvieron estacionarias. El grupo de causas evitables presentó mayores tasas de mortalidad en ambas clasificaciones, con tendencia creciente según LBE-OF (Variación Porcentual Anual-APC: 2,1; p = 0,018) y estacionaria según LBE < 5. Hubo tendencia decreciente para la mortalidad por causas mal definidas sólo según LBE-OF (APC: –12,3; p < 0,001).

**Conclusión:**

Los resultados mostraron el estancamiento de la tendencia temporal de la mortalidad fetal, la evitabilidad de la mayoría de las muertes y el potencial del LBE-OF para monitorear la calidad de la información sobre las causas básicas y la evitabilidad de las muertes fetales.

## INTRODUCTION

Fetal mortality remains a serious global public health problem, largely neglected and unevenly distributed^(1)^. An estimated 84% of the global burden is concentrated in low- and middle-income nations^(2)^. In Brazil, between 1996 and 2019, the fetal mortality rate (FMR) was reduced by 25.2%, from 13.5 to 10.1 per 1,000 total births (TB)^(3)^. In the country, fetal deaths have a heterogeneous distribution, with higher rates in the Northeast region, which are higher than the national average^(3,4)^. However, the city of Recife, Pernambuco (PE), has recorded rates lower than the national ones and those of their respective state and region^(3)^. These variations in health indicators highlight the importance of local analyses for understanding fetal mortality and for planning effective interventions that are appropriate to the reality of each population^(4,5)^.

Understanding fetal mortality involves not only counting, but also investigating the causes and avoidability of deaths^(6,7)^. In this sense, the importance of avoidability classification systems stands out, which allow the identification of avoidable deaths and health actions that could prevent these outcomes^(8,9)^.

In Brazil, among the known avoidability classifications for fetal death are the Expanded Wigglesworth (adapted for Brazil) and the most recent Brazilian List of Avoidable Causes of Death for fetal deaths (LBE-OF)^(7,10)^. However, avoidability classifications developed for infant deaths are also applied to fetal deaths, such as the Brazilian List of Causes of Avoidable Deaths for children under five years of age (LBE < 5)^(5,9,10)^.

Classifications consistent with the pathophysiology of fetal death are essential to understand the potential for preventing these deaths and identify ill-defined causes, which present a high percentage throughout the world^(10,11)^. From this perspective, the objective of this study was to analyze the temporal trend of fetal mortality and its components, of preventable and ill-defined causes according to two avoidability classifications in Recife, Pernambuco, 2010–2021.

## METHOD

### Type of Study

Ecological study of temporal trends, whose analysis units were the years between 2010 and 2021.

### Location and Period of Study

The area of study was Recife, capital of the state of Pernambuco, located in the Northeast region of Brazil. The city is the 17th most populous in the country, with an estimated population of 1,488,920 people^(12)^. In Recife, the Mortality Information System (MIS) and the Live Birth Information System (Sinasc in the acronym in Portuguese) are of good quality, and the performance of death surveillance, based on corrections to vital statistics, has contributed to the consolidation of these information systems^(13)^. Therefore, to delimit the selected period (2010 to 2021), Ordinance No. 72 of 2010 of the Ministry of Health was taken into consideration, which establishes mandatory fetal death surveillance, and the last year with data available on MIS and Sinasc. The time series also included the implementation of Rede Cegonha (2011)^(14)^ and the COVID-19 pandemic (2020 and 2021).

### Population and Data Acquisition

Information on fetal deaths (gestational age greater than or equal to 22 weeks and/or birth weight greater than or equal to 500 grams) and the number of live births to mothers residing in Recife were used. The research data came from MIS and Sinasc, obtained from the Epidemiological Surveillance of the Recife Health Department.

### Data Analysis and Processing

Fetal deaths were classified as early and late, considering, respectively: gestational age greater than or equal to 22 and less than 28 weeks or, in the absence of this information, birth weight greater than or equal to 500 and less than 1,000 grams; and gestational age greater than or equal to 28 weeks or, in the absence of this information, birth weight greater than or equal to 1,000 grams.

Of the 2,490 fetal deaths recorded, 40 (1.6%) had missing information on gestational age and birth weight, which could not be classified as early or late, and were not included in the temporal trend of fetal mortality components.

The avoidability analysis was carried out using the Brazilian List of Causes of Avoidable Deaths for fetal deaths (LBE-OF)^(10)^ and for children under 5 years of age (LBE < 5)^(9)^. The two lists classify deaths according to the underlying cause of death, according to the International Classification of Diseases and Related Health Problems, 10th Revision (ICD-10), into three groups: avoidable causes, ill-defined causes of death, and other causes (not clearly avoidable).

In LBE-OF, fetal deaths from avoidable causes are classified into the subgroups of health actions: immunoprevention; adequate care for women during pregnancy; and adequate care for women during childbirth^(10)^. In LBE < 5, the following are added: adequate care for the fetus and newborn; appropriate diagnostic and treatment actions; and appropriate health promotion and health care actions^(9)^.

The differences between LBE-OF and LBE < 5, in addition to the subgroups of avoidable causes, refer to the organization of basic causes between groups and subgroups^(9,10)^. In the study, based on the temporal aggregate from 2010 to 2021, the proportion of fetal deaths by group and subgroup of avoidability of LBE-OF and LBE < 5 was calculated.

McNemar’s test for paired samples (before and after) was applied to evaluate differences between the avoidability categories of the two lists used. The before, the groups and subgroups of LBE < 5 were considered, after, the LBE-OF, assuming a significance level of 5% for all analyses. Data were analyzed using *Minitab Statistical Software*, version 21.4.0.

To analyze the temporal trend, the total, early, late, avoidable and ill-defined fetal mortality rates were calculated, according to the two classifications and by year of occurrence. For the calculation, the number of fetal deaths divided by the total number of births multiplied by 1,000 was considered.

Temporal trends in fetal mortality were analyzed using the *Joinpoint* regression model, *Joinpoint* program, version 4.9.1.0, which tests whether a multi-segmented line is statistically better at describing the temporal evolution of the analyzed event than a straight line or with less segments^(15)^. The model checks whether changes in the trend are statistically significant, and allows them to be classified as increasing, decreasing or stationary, based on the significance test and the annual percentage variation (APC: annual percent change)^(15,16)^.

The program also informs the average annual percentage change for the period analyzed (AAPC: *average annual percent change*), which refers to the summary measure of the trend, calculated from the weighted average of the APC^(16)^. The AAPC allows you to compare non-constant trends in different groups over the period of interest^(16)^. In the analysis, fetal mortality rates corresponded to the dependent variables and the years corresponded to the independent variables. The level of statistical significance of p < 0.05 was admitted.

### Ethical Aspects

The research was developed based on secondary data, and is in accordance with Resolution 466/2012 of the National Health Council, and was approved by the Research Ethics Committee of the Federal University of Pernambuco in 2022, under Opinion 6,317,405.

## RESULTS

**Figure 1:**
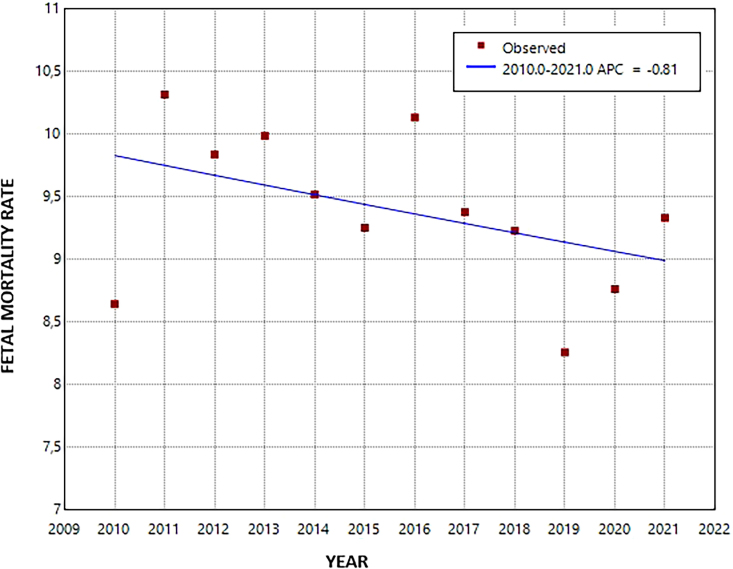
Temporal trend in fetal mortality. Recife, Pernambuco, 2010–2021.

APC: annual percentage change; Source: Mortality Information System (MIS) and Live Birth Information System (Sinasc).

During the period studied, there were 2,490 fetal deaths and 262,300 live births, totaling 264,790 TB of mothers living in Recife. The initial FMR (2010) was 8.6 and the final (2021) was 9.3 per 1,000 TB (Figure 1). Of the total deaths, 1,874 (75.3%) were late deaths. There was a stationary temporal trend for fetal mortality and its early and late components (Table 1).

**Table 1:** Temporal trend of fetal mortality and its early and late components. Recife, Pernambuco, Brazil, 2010–2021.

Mortality rates (per 1,000 total births)	FMR		Estimates			*p-value	Trend
	2010	2021	APC	IC 95%			
Total fetal	8,6	9,3	–0,8	–2,0	0,4	**0,163**	**Stationary**
Early fetal	2,1	2,3	–1,2	–3,9	1,5	**0,336**	**Stationary**
Late fetal	6,1	7,0	–0,2	–1,8	1,4	**0,758**	**Stationary**

APC: annual percentage change; CI95%: 95% confidence interval;

*Joinpoint Regression Model; FMR: fetal mortality rate per thousand total births.

Source: Mortality Information System (MIS) and Live Birth Information System (Sinasc).

According to the LBE-OF, there were 1,814 avoidable fetal deaths (72.9%), 471 due to ill-defined causes (18.9%) and 205 (8.2%) due to causes that were not clearly avoidable. With LBE < 5, 2,091 (84.0%) avoidable fetal deaths were classified, 205 (8.2%) due to ill-defined causes and 194 (7.8%) due to causes that were not clearly avoidable (Table 2).

**Table 2:** Avoidability of fetal deaths (n = 2,490) according to the Brazilian List of Avoidable Causes of Death for fetal deaths (LBE-OF) and the Brazilian List of Avoidable Causes of Death for children under 5 years of age (LBE < 5). Recife, Pernambuco, Brazil, 2010–2021.

Classification of avoidability of causes of death	LBE < 5		LBE-OF		p-value*
	n	%	n	%	
1. Avoidable causes	2.091	84,0	1.814	72,9	< 0,001
1.1 Reducible by immunoprevention actions	0	–	0	–	
1.2. Reducible by adequate care for women during pregnancy, childbirth and the newborn					
1.2.1. Reducible by adequate care for women during pregnancy	1.355	64,8	1.447	79,8	< 0,001
1.2.2. Reducible by adequate care for women during childbirth	595	28,5	367	20,2	< 0,001
1.2.3. Reducible by adequate care for the fetus and newborn	140	6,7	NA	NA	
1.3. Reducible by appropriate diagnostic and treatment actions	0	–	NA	NA	
1.4. Reducible by appropriate health promotion and health care actions	1	0,0	NA	NA	
2. Ill-defined causes of death	205	8,2	471	18,9	< 0,001
3. Other causes (not clearly avoidable)	194	7,8	205	8,2	0,001
Total	2.490	100	2.490	100	

NA: not applicable;

*McNemar test.

Source: Mortality Information System (MIS) and Live Birth Information System (Sinasc).

Among the avoidable deaths, in both classifications, those that could be reduced by adequate care for women during pregnancy predominated, with 1,447 (79.8%) according to LBE-OF and 1,355 (64.8%) according to LBE < 5. The subgroup that can be reduced by adequate care for women during childbirth presented 367 (20.2%) deaths when using the LBE-OF classification, and 595 (28.5%) deaths with LBE < 5 (Table 2).

For LBE < 5, causes that can be reduced by adequate care for the fetus and newborn accounted for 141 (6.7%) fetal deaths, and causes that can be reduced by adequate health promotion actions, linked to adequate care actions for the health for 1 (0.05%) fetal death (Table 2).

Considering the entire study period, avoidable fetal mortality showed an increasing temporal trend according to LBE-OF (APC: 2.1; p = 0.018) and a stationary temporal trend according to LBE < 5 years (Table 3).

**Table 3:** Temporal trend in fetal mortality from avoidable and ill-defined causes according to the Brazilian List of Avoidable Causes of Death for fetal deaths (LBE-OF) and the Brazilian List of Avoidable Causes of Death for children under 5 years of age (LBE < 5). Recife, Pernambuco, Brazil, 2010–2021.

Mortality rates (per 1,000 total births)	Trending Periods	FMR		Estimates			**p–Value	Estimates			**p–value	Trend
		Start	End	APC	IC 95%			AAPC	IC 95%			
**Brazilian List of Avoidable Causes of Death for fetal deaths (LBE–OF)**												
Avoidable fetal	2010–2021*	5,6	7,4	2,1*	0,4	3,8	**0,018**					**Increasing**
*Fetal reducible by adequate care for women during pregnancy*	2010–2016*	3,9	6,6	7,9*	1,7	14,5	**0,019**	2,7	–1,2	6,8	**0,171**	**Stationary**
	2016–2021	6,6	5,8	–3,1	–10,1	4,4	**0,348**					
*Fetal reducible by adequate care for women during childbirth*	2010–2016*	1,6	0,9	–11,2*	–20,6	–0,7	**0,041**	–0,1	–7,7	8,2	**0,984**	**Stationary**
	2016–2021	0,9	1,6	15,1	–2,1	35,3	**0,079**					
Ill-defined fetal	2010–2021*	2,5	1,1	–12,3*	–16,9	–7,4	**< 0,001**					**Decreasing**
**Brazilian List of Avoidable Causes of Death for children under 5 years of age (LBE < 5)**												
Avoidable fetal	2010–2021	7,2	7,6	–0,3	–1,7	1,2	**0,687**					**Stationary**
*Fetal reducible by adequate care for women during pregnancy*	2010–2016*	3,7	6,2	7,5*	0,7	14,9	**0,035**	2,0	–2,3	6,5	**0,363**	**Stationary**
	2016–2021	6,2	5,5	–4,2	–11,9	4,1	**0,261**					
*Fetal reducible by adequate care for women during childbirth*	2010–2018*	3,1	1,6	–8,7*	–11,1	–6,3	**< 0,001**	–5,8*	–9,3	–2,1	**0,002**	**Decreasing**
	2018–2021	1,6	1,6	2,6	–12,1	19,7	**0,711**					
*Fetal reducible by adequate care for the fetus and newborn*	2010–2021	0,4	0,5	5,2	–0,9	11,7	**0,088**					**Stationary**
Ill-defined fetal	2010–2012	0,9	1,4	19,8	–44,3	158,0	**0,548**	0,2	–16,0	19,4	**0,985**	**Stationary**
	2012–2019*	1,4	0,2	–19,5*	–31,5	–5,3	**0,021**					
	2019–2021	0,2	0,9	79,7	–32,5	378,8	**0,172**					

APC: annual percentage change; AAPC: average annual percentage change; CI95%: 95% confidence interval;

*p < 0,05;

** Joinpoint Regression Model; FMR: fetal mortality rate per thousand total births.

Source: Mortality Information System (MIS) and Live Birth Information System (Sinasc).

For both classifications, the temporal trend of deaths that can be reduced by adequate care for women during pregnancy was increasing between 2010 and 2016 (APC: 7,9; p = 0,019; APC: 7,5; p = 0,035) and stationary between 2016 and 2021. For deaths that can be reduced by adequate care for women during childbirth, there was a decreasing temporal trend in overlapping periods: according to the LBE-OF, between 2010 and 2016 (APC: –11,2; p = 0,041), and LBE < 5 years, between 2010 and 2018 (APC: –8,7; p < 0,001). This decreasing period was followed by a stationary temporal trend according to both classifications. Considering the total period, there was a stationary trend according to LBE-OF, and a decreasing trend for LBE < 5 years (AAPC: –5,8; p < 0,002). Regarding deaths that can be reduced by adequate care for the fetus and newborn, LBE < 5 years, the trend was stationary throughout the period analyzed (Table 3).

There was a decreasing temporal trend for fetal mortality due to ill-defined causes according to the LBE-OF (APC: –12.3; p < 0.001). For LBE < 5 years, three temporal trends were identified: stationary, between 2010 and 2012, decreasing between 2012 and 2019 (APC: –19.5; p = 0.021) and stationary between 2019 and 2021. Considering the total period of the series , the temporal trend was stationary for fetal mortality from ill-defined causes according to LBE < 5 years (Table 3).

## DISCUSSION

Fetal mortality in Recife showed a stationary temporal trend between 2010 and 2021, without significant variations, even during the years of the COVID-19 pandemic. Early and late fetal mortality showed a stationary trend, with a predominance of late fetal deaths, which corresponded to more than two thirds of fetal deaths.

The finding that FMR in Recife has not shown a significant decline for more than a decade, although it is lower than the rates observed in Pernambuco, the Northeast region and Brazil as a whole, reveals the need for improvements in prenatal and intrapartum care^(4,5)^. Similar to Recife, other studies carried out in the country found a stationary temporal trend in fetal mortality: in Brazil, between 1996 and 2015; in all regions of the country, between 2000 and 2015; and in the city of São Paulo, from 2007 to 2017^(4,17)^.

These results contrast with the decrease in neonatal and infant mortality observed in different areas of Brazil, including Recife^(18,19,20)^. Women’s and children’s health actions, mainly in primary health care with the expansion of the Family Health Strategy, have contributed to a decrease in infant mortality rates in the last two decades^(21)^.

Despite the reduction in neonatal and infant mortality rates suggesting advances in maternal and child health, the need for additional investments to prevent fetal deaths, especially late deaths, is confirmed^(1)^. The stationary trend in fetal mortality found in this study reaffirms this need. The invisibility of fetal death in relation to infant death occurs not only in Brazil, but on the international scene, considering that fetal mortality was not included in the Millennium Development Goals (MDG) agenda and is not present in the Sustainable Development Goals (SDG)^(2)^

It is noteworthy that *Rede Cegonha* was implemented in Brazil, in 2011, as a strategy to ensure improved access, coverage and quality of prenatal care and childbirth, postpartum and childcare^(14)^. However, an assessment of the degree of implementation of good practices in labor and birth care in *Rede Cegonha* maternities indicates that, despite the advances achieved, there is a long way to go to qualify these health services^(14)^.

In relation to the COVID-19 pandemic, in Brazil and around the world, few time series studies have been carried out to analyze the impact of this event on the trend in fetal mortality^(22,23)^. The present study observed a stationary trend in fetal mortality in Recife during the years of 2020 and 2021, similar to that found for Alabama, in the United States^(22)^. In contrast, research carried out in Mozambique identified a decreasing trend in fetal mortality during the pandemic period^(23)^.

Studies conducted to investigate possible differences in the number of fetal deaths during the COVID-19 pandemic, compared to the pre-pandemic period, found discordant results^(22,23,24,25)^. These findings have been explained by factors associated with the risks related to COVID-19 during pregnancy, the socioeconomic and health conditions of the population, and the restrictions on access to healthcare imposed during this period^(24,25)^.

The predominance of late fetal deaths evidenced in Recife is in agreement with what occurs in Brazil and regions^(3)^. Differently, in high-income countries, most fetal deaths are early and relatively more difficult to prevent, with congenital anomalies among the main causes of death^(4,26)^.

It is noteworthy that in 2021, the late FMR in Recife (7.0 per 1,000 TB) was higher than that in the city of São Paulo (5.0), and close to that in the city of Rio de Janeiro (6.8) and that of Brazil (6.7)^(3)^. The comparison with developed countries, such as Sweden, which in 2019 had 2.5 late deaths per 1,000 TB, highlights disparities in fetal mortality and the potential for avoidng these deaths^(26)^.

Regarding the avoidability of fetal deaths that occurred in Recife between 2010 and 2021, most of them were considered avoidable according to both classifications. This result is in agreement with studies carried out in Brazil and other low- and middle-income countries, in which the majority of conditions related to fetal deaths are avoidable through the quality of prenatal and intrapartum care^(2,5,10,20)^.

The use of LBE < 5 years resulted in an 11.1% higher frequency of avoidable deaths compared to LBE-OF. This result could suggest a greater sensitivity of LBE < 5 years in identifying the avoidability of fetal deaths, however, it is due to the inclusion of causes considered poorly defined in the subgroups that can be reduced by adequate care for women during childbirth and reducible by adequate care for the fetus and the newborn.

Among the avoidability subgroups, those that can be reduced by adequate care for women during pregnancy presented the highest frequency of fetal deaths according to both lists. At the same time, national studies point to the avoidability of most fetal deaths, mainly through adequate pregnancy care, followed by adequate birth care^(5,10,20)^.

The finding that most fetal deaths could have been avoided, mainly through adequate care during pregnancy, points to deficiencies in this health care. In Brazil, the assessment of the adequacy of prenatal care highlighted weaknesses in access, coverage and quality of this service. Reaffirming the need for government actions to guarantee quality prenatal care^(27)^.

The research that compared the results of LBE < 5 years and LBE-OF in the classification of 2,585 fetal deaths that occurred in 2018 in the state of Rio de Janeiro found percentages of 6.7% and 35.5% for the group of ill-defined causes, respectively^(10)^. Accordingly, in the present study, when using LBE-OF, the group of ill-defined causes presented a higher percentage of deaths compared to LBE < 5 years.

These results are explained by differences in the allocation of some ill-defined causes. Avoidability classifications consistent with the pathophysiology of fetal death are important to assess the real potential for avoiding deaths, communicate weaknesses in the attribution of causes, and indicate the need for improvements in the availability and/or quality of *postmortem* exams^(10,28)^.

In Brazil, 36.6% of fetal deaths occurring between 2013 and 2016 had as their underlying cause unspecified intrauterine hypoxia (ICD-10 P20.9) or fetal death of unspecified cause (ICD-10 P95)^(29)^. In the city of São Paulo, a study that analyzed the basic causes of 720 fetal deaths recorded between 2012 and 2014, identified these as the causes of 44.7% of deaths^(17)^. In the city of Rio de Janeiro, ill-defined causes accounted for 25.8% of fetal deaths recorded in 2018^(20)^. Considering the results of these studies, which classified unspecified intrauterine hypoxia (ICD-10 P20.9) in the group of ill-defined causes, it is suggested that in Recife there is a lower percentage of these causes, which may be related, among other factors, the effectiveness of death surveillance^(18)^.

The analysis of temporal trends in avoidable fetal mortality according to LBE < 5 years and LBE-OF revealed distinct trends, increasing according to LBE-OF and stationary according to LBE < 5 years. It is possible that the increase in avoidable fetal mortality according to the LBE-OF is related to the decreasing trend in ill-defined causes. Therefore, with the improvement in the attribution of causes, a greater number of deaths began to be classified as avoidable. In contrast, for LBE < 5 years, when considering the total period of the series, temporal trends in avoidable and ill-defined fetal mortality remained stationary, suggesting that this list was not able to detect all the progress made in attributing causes basic. However, the decreasing trend detected in fetal mortality due to ill-defined causes according to the LBE < 5 years, in the period from 2012 to 2019, reaffirms the importance of death surveillance for the qualification of causes of death in the municipality.

Considering fetal mortality that can be reduced by adequate care during pregnancy, in the total period of the series, a stationary trend was observed according to both classifications, which confirms the need for investments that guarantee, in addition to early capture and the minimum number of six consultations, the quality of these services^(27)^. Similar to developed countries, the results showed a higher fetal mortality rate related to the quality of pregnancy care to the detriment of childbirth care^(2)^.

When considering the total study period, the stationary trend of the adequate birth care component according to the LBE-OF highlights the importance of continuous improvements in intrapartum care. In contrast, according to LBE < 5 years, when considering the total period of the series, there was a decreasing trend in fetal mortality reducible by adequate care during childbirth. It is suggested that this difference is related to the decrease in the attribution of the cause of unspecified intrauterine hypoxia (ICD-10 P20.9), classified in LBE < 5 years as reducible by adequate care during childbirth.

In Brazil and around the world, hypoxia has been frequently reported as a cause of fetal deaths when, in fact, it refers to the pathological pathway through which death occurs^(6,11,29)^. Hypoxia, like other ill-defined causes, is not useful for identifying the determining factors of death and does not contribute to the planning of interventions and health policies^(6,11)^.

Understanding what favors the attribution of ill-defined causes is the first step to intervening in their reduction. This may be related to the poor quality of clinical records, poor training of professionals to identify the underlying cause and the unavailability or low quality of *postmortem* examinations^(6,11)^. To a lesser extent, ill-defined causes are associated with unexplained fetal deaths, for which, even after adequate *postmortem* examinations, the causes have not been clarified^(11,30)^.

Regarding the limitations of this study, the use of secondary data is highlighted, which may contain incomplete records and gaps in coverage. However, the completeness and agreement of Recife’s Information Systems on Mortality and Live Births are considered adequate, which confirms their applicability for measuring the population’s health situation^(13)^.

## CONCLUSION

The stagnation of the trend in fetal mortality for more than a decade and the avoidability of most fetal deaths, mainly through adequate prenatal care and childbirth, reveal the need to improve prenatal care and continuous investments in intrapartum care. Qualified professionals in adequate numbers, access to timely prenatal exams, classification of gestational risk, appropriate management of high-risk pregnancies, facilities and material resources to offer quality care are essential to achieving a reduction in fetal deaths.

The differences found in the classification of avoidability of fetal deaths according to the LBE < 5 years and the LBE-OF, mainly the higher frequency of deaths classified in the group of ill-defined causes when using the LBE-OF, highlights the potential of this classification in monitoring the quality of information on the causes of fetal deaths. Therefore, the use of LBE-OF in fetal death surveillance can contribute to the planning of actions that favor the correct identification of the basic causes of death and, therefore, to the reduction of poorly defined causes.

The decreasing temporal trend of ill-defined causes, evidenced when using the LBE-OF, reveals the advances achieved in attributing the basic causes of fetal deaths in Recife, which suggests the effectiveness of fetal death surveillance and reinforces the importance of this service and investments that promote its strengthening. This result confirms the possibility of other cities also reducing ill-defined causes.

## References

[1] Hug L, You D, Blencowe H, Mishra A, Wang Z, Fix M (2021). Global, regional, and national estimates and trends in stillbirths from 2000 to 2019: a systematic assessment.. Lancet..

[2] You D, Hug L, Mishra A, Blencowe H, Moran A (2020). A neglected tragedy: the global burden of stillbirths [Internet]..

[3] Brasil. (2022). Ministério da Saúde. Datasus.. Estatísticas vitais [Internet]..

[4] Barros PS, Aquino EC, Souza MR (2019). Mortalidade fetal e os desafios para a atenção à saúde da mulher no Brasil.. Rev Saude Publica..

[5] Canuto IM, Macêdo VC, Frias PG, Oliveira CM, Bonfim CV (2021). Perfil epidemiológico, padrões espaciais e evitabilidade da mortalidade fetal em Pernambuco.. Acta Paul Enferm..

[6] Aminu M, van den Broek N (2019). Stillbirth in low-and middle-income countries: addressing the ‘silent epidemic’.. Int Health..

[7] Brasil. (2023). Ministério da Saúde. Secretaria de Vigilância em Saúde. Secretaria de Atenção à Saúde.. Manual de vigilância do óbito infantil e fetal e do Comitê de Prevenção do Óbito Infantil e Fetal [Internet]. Brasília: Ministério da Saúde;.

[8] Leisher SH, Teoh Z, Reinebrant H, Allanson E, Blencowe H, Erwich JJ (2016). Seeking order amidst chaos: a systematic review of classification systems for causes of stillbirth and neonatal death, 2009–2014.. BMC Pregnancy Childbirth..

[9] Malta DC, Sardinha LMV, Moura L, Lansky S, Leal MC, Szwarcwald CL (2010). Atualização da lista de causas de mortes evitáveis por intervenções do Sistema Único de Saúde do Brasil.. Epidemiol Serv Saude..

[10] Fonseca SC, Kale PL, Teixeira GHMC, Lopes VGS (2021). Evitabilidade de óbitos fetais: reflexões sobre a Lista Brasileira de Causas de Mortes Evitáveis por intervenção do Sistema Único de Saúde.. Cad Saude Publica..

[11] Reinebrant HE, Leisher SH, Coory M, Henry S, Wojcieszek AM, Gardener G (2018). Making stillbirths visible: a systematic review of globally reported causes of stillbirth.. BJOG..

[12] Instituto Br asileiro de Geografia e Estatística. (2022). Cidades [Internet]..

[13] Romaguera AA, Guimarães ALS, Oliveira CM, Cardoso MD, Bonfim CV (2020). Concordância e completude dos dados sobre nascidos vivos e óbitos infantis.. Acta Paul Enferm..

[14] Bittencourt SDA, Vilela MEA, Marques MCO, Santos AM, Silva CKRT, Domingues RMSM (2021). Atenção ao parto e nascimento em Maternidades da Rede Cegonha/Brasil: avaliação do grau de implantação das ações.. Cien Saude Colet..

[15] Kim HJ, Fay MP, Feuer EJ, Midthune DN (2000). Permutation tests for joinpoint regression with applications to cancer rates.. Stat Med..

[16] Clegg LX, Hankey BF, Tiwari R, Feuer EJ, Edwards BK (2009). Estimating average annual percent change in trend analysis.. Stat Med..

[17] Marques LJP, Silva ZP, Alencar GP, Almeida MF (2021). Contribuições da investigação dos óbitos fetais para melhoria da definição da causa básica do óbito no Município de São Paulo, Brasil.. Cad Saude Publica..

[18] Oliveira CM, Bonfim CV, Guimarães MJB, Frias PG, Medeiros ZM (2016). Mortalidade infantil: tendência temporal e contribuição da vigilância do óbito.. Acta Paul Enferm..

[19] Bernardino FBS, Gonçalves TM, Pereira TID, Xavier JS, Borges BH, Gaíva MAM (2022). Tendência da mortalidade neonatal no Brasil de 2007 a 2017.. Cien Saude Colet..

[20] Kale PL, Fonseca SC, Oliveira PWM, Brito AS (2021). Tendência da mortalidade fetal e infantil segundo evitabilidade das causas de morte e escolaridade materna.. Rev Bras Epidemiol..

[21] Hatisuka MFB, Moreira RC, Cabrera MAS (2021). Relação entre a avaliação de desempenho da atenção básica e a mortalidade infantil no Brasil.. Cien Saude Colet..

[22] Shukla VV, Rahman AKMF, Shen X, Black A, Arora N, Lal CV (2023). Trends in fetal and neonatal outcomes during the COVID-19 pandemic in Alabama.. Pediatr Res..

[23] Lydon MM, Vilanculos J, Martinez A, Barata A, Keyes E (2022). Effects of the COVID-19 pandemic on maternal and perinatal health service utilisation and outcomes in Mozambique: an interrupted time series analysis.. BMJ Open..

[24] Handley SC, Mullin AM, Elovitz MA, Gerson KD, Montoya-Williams D, Lorch SA (2021). Changes in preterm birth phenotypes and stillbirth at 2 Philadelphia Hospitals During the SARS-CoV-2 Pandemic, March-June 2020.. JAMA..

[25] Khalil A, von Dadelszen P, Draycott T, Ugwumadu A, O’Brien P, Magee L (2020). Change in the incidence of stillbirth and preterm delivery during the COVID-19 Pandemic.. JAMA..

[26] Euro-Peristat. (2020). European Perinatal Health Report.. Core indicators of the health and care of pregnant women and babies in Europe from 2015 to 2019 [Internet]..

[27] Luz LA, Aquino R, Medina MG (2018). Avaliação da qualidade da Atenção Pré-Natal no Brasil.. Saúde Debate..

[28] Pekkola M, Tikkanen M, Loukovaara M, Lohi J, Paavonen J, Stefanovic V (2020). Postmortem examination protocol and systematic re-evaluation reduce the proportion of unexplained stillbirths.. J Perinat Med..

[29] Ministério da Saúde (BR). (2019). Secretaria de Vigilância em Saúde. Departamento de Vigilância de Doenças e Agravos não Transmissíveis e Promoção da Saúde.. Saúde Brasil 2018 uma análise de situação de saúde e das doenças e agravos crônicos: desafios e perspectivas [Internet]. Brasília: Ministério da Saúde;.

[30] Frøen JF, Gordijn SJ, Abdel-Aleem H, Bergsjo P, Betran A, Duke CW (2009). Making stillbirths count, making numbers talk - issues in data collection for stillbirths.. BMC Pregnancy Childbirth..

