# Supplementary material for: Temporal trend in fetal mortality according to two death avoidability classifications
Source: Rev Esc Enferm USP. 2024 Sep 27;58:e20240015. doi: 10.1590/1980-220X-REEUSP-2024-0015en (PMC11433960; doi:10.1590/1980-220X-REEUSP-2024-0015en)
Supplement: Translation

# Translation: Tendência temporal da mortalidade fetal segundo duas classificações de evitabilidade do óbito

**DOI:** 10.1590/1980-220X-REEUSP-2024-0015pt

**Authors:** Rebeca Danielly Barros Xavier, Mirella Bezerra Rodrigues Vilela, Cristine Vieira do Bonfim, Conceição Maria Oliveira, Pedro Israel Cabral de Lira, Silvia Wanick Sarinho

**Affiliations:** 1 Secretaria de Estado da Saúde de Alagoas, Hospital da Mulher Dra. Nise da Silveira, Maceió, AL, Brasil.; 2 Universidade Federal de Pernambuco, Departamento de Fonoaudiologia, Recife, PE, Brasil.; 3 Fundação Joaquim Nabuco, Diretoria de Pesquisas Sociais. Recife, PE, Brasil.; 4 Secretaria de Saúde do Recife, Secretaria Executiva de Vigilância à Saúde. Recife, PE, Brasil.; 5 Universidade Federal de Pernambuco, Centro de Ciências da Saúde, Departamento de Nutrição. Recife, PE, Brasil.; 6 Universidade Federal de Pernambuco, Programa de Pós‐Graduação em Saúde da Criança e do Adolescente. Recife, PE, Brasil.

**Keywords:** Mortalidade Fetal, Causas de Morte, Monitoramento Epidemiológico, Estudos de Séries Temporais

## Abstract

**Objetivo:**

Analisar a tendência temporal da mortalidade fetal e de seus componentes, das causas evitáveis e mal definidas segundo duas classificações de evitabilidade no Recife, Pernambuco, 2010–2021.

**Método:**

Estudo ecológico de tendência temporal para a mortalidade fetal no Recife, 2010–2021. Foram utilizadas as classificações de evitabilidade Lista Brasileira de Causas de Morte Evitáveis para óbitos fetais (LBE-OF) e Lista Brasileira de Causas de Morte Evitáveis para menores de cinco anos (LBE < 5). O modelo de regressão *Joinpoint* foi aplicado para analisar as tendências temporais.

**Resultados:**

As tendências da mortalidade fetal e de seus componentes foram estacionárias. O grupo de causas evitáveis apresentou maiores taxas de mortalidade nas duas classificações, com tendência crescente segundo a LBE-OF (Variação Percentual Anual-APC: 2,1; p = 0,018) e estacionária segundo a LBE < 5. Houve tendência decrescente para a mortalidade por causas mal definidas apenas conforme a LBE-OF (APC: –12,3; p < 0,001).

**Conclusão:**

Os resultados evidenciaram a estagnação da tendência temporal da mortalidade fetal, a evitabilidade da maior parte dos óbitos, e a potencialidade da LBE-OF no monitoramento da qualidade das informações sobre as causas básicas e evitabilidade dos óbitos fetais.

## INTRODUÇÃO

A mortalidade fetal persiste como um grave problema de saúde pública mundial, amplamente negligenciado e com distribuição desigual^(1)^. Estima-se que 84% da carga global concentra-se em nações de baixa e média renda^(1)^. No Brasil, entre 1996 e 2019, a taxa de mortalidade fetal (TMF) alcançou redução de 25,2%, passando de 13,5 para 10,1 por 1.000 nascimentos totais (NT)^(1)^. No país, os óbitos fetais apresentam distribuição heterogênea, com maiores taxas na região Nordeste, as quais são superiores à média nacional^(1,3)^. Entretanto, a cidade do Recife, Pernambuco (PE), tem registrado taxas inferiores às nacionais e às dos seus respectivos estado e região^(1)^. Essas variações nos indicadores de saúde destacam a importância das análises locais para compreensão da mortalidade fetal e para o planejamento de intervenções eficazes e adequadas à realidade de cada população^(1,4)^.

O entendimento sobre a mortalidade fetal envolve não apenas a contagem, mas também a averiguação das causas e da evitabilidade dos óbitos^(1,6)^. Nesse sentido, destaca-se a importância dos sistemas de classificação de evitabilidade, que permitem identificar os óbitos evitáveis e as ações de saúde que poderiam prevenir esses desfechos^(1,8)^.

No Brasil, entre as classificações de evitabilidade para o óbito fetal conhecidas, estão a Wigglesworth Expandida (adaptada para o Brasil) e a mais recente Lista Brasileira de Causas de Mortes Evitáveis para óbitos fetais (LBE-OF)^(1,7)^. Entretanto, classificações de evitabilidade desenvolvidas para óbitos infantis também são aplicadas para as mortes fetais, como a Lista Brasileira de Causas de Mortes Evitáveis para menores de cinco anos (LBE < 5)^(5,9,10)^.

Classificações condizentes com a fisiopatologia do óbito fetal são essenciais para entender o potencial de prevenção desses óbitos e identificar as causas mal definidas, as quais apresentam elevado percentual em todo o mundo^(1,10)^. Nessa perspectiva, o objetivo deste estudo foi analisar a tendência temporal da mortalidade fetal e de seus componentes, das causas evitáveis e mal definidas segundo duas classificações de evitabilidade no Recife, Pernambuco, 2010-2021.

## MÉTODO

### Tipo de Estudo

Estudo ecológico de tendência temporal, cujas unidades de análises foram os anos compreendidos entre 2010 e 2021.

### Local e Período do Estudo

A área do estudo foi o Recife, capital do estado de Pernambuco, localizada na região Nordeste do Brasil. A cidade é a 17ª mais populosa do país, com uma população estimada em 1.488.920 pessoas^(1)^. No Recife, o Sistema de Informações sobre Mortalidade (SIM) e o Sistema de Informações sobre Nascidos Vivos (Sinasc) apresentam boa qualidade, e a atuação da vigilância do óbito, a partir das correções das estatísticas vitais, tem contribuído para a consolidação desses sistemas de informações^(1)^. Assim sendo, para a delimitação do período selecionado (2010 a 2021), levou-se em consideração a Portaria nº 72 de 2010 do Ministério da Saúde, que institui a vigilância do óbito fetal obrigatória, e o último ano com dados disponíveis no SIM e Sinasc. A série temporal também incluiu a implantação da Rede Cegonha (2011)^(1)^ e a pandemia pela covid-19 (2020 e 2021).

### População e Obtenção dos Dados

Foram utilizadas as informações sobre os óbitos fetais (idade gestacional maior ou igual a 22 semanas e/ou peso ao nascer maior ou igual a 500 gramas) e o número de nascidos vivos de mães residentes no Recife. Os dados da pesquisa foram provenientes do SIM e do Sinasc, obtidos na Vigilância Epidemiológica da Secretaria de Saúde do Recife.

### Análise e Tratamento dos Dados

Os óbitos fetais foram classificados em precoce e tardio, considerando, respectivamente: idade gestacional maior ou igual a 22 e menor que 28 semanas ou, na ausência dessa informação, peso ao nascer maior ou igual a 500 e menor que 1.000 gramas; e idade gestacional maior ou igual a 28 semanas ou, na ausência dessa informação, peso ao nascer maior ou igual a 1.000 gramas.

Dos 2.490 óbitos fetais registrados, 40 (1,6%) tinham informações ignoradas sobre a idade gestacional e o peso ao nascer, os quais não puderam ser classificados em precoces ou tardios, e não foram incluídos na tendência temporal dos componentes da mortalidade fetal.

A análise da evitabilidade foi realizada por meio da Lista Brasileira de Causas de Mortes Evitáveis para óbitos fetais (LBE-OF)^(1)^ e para menores de 5 anos (LBE < 5)^(1)^. As duas listas classificam os óbitos conforme a causa básica da morte, segundo a Classificação Internacional de Doenças e Problemas Relacionados à Saúde, 10ª Revisão (CID-10), em três grupos: causas evitáveis, causas de mortes mal definidas, e demais causas (não claramente evitáveis).

Na LBE-OF, os óbitos fetais por causas evitáveis são classificados nos subgrupos de ações de saúde: imunoprevenção; adequada atenção à mulher na gestação; e adequada atenção à mulher no parto^(1)^. Na LBE < 5, acrescentam-se: adequada atenção ao feto e ao recém-nascido; ações adequadas de diagnóstico e tratamento; e ações adequadas de promoção à saúde e atenção à saúde^(1)^.

As diferenças entre a LBE-OF e a LBE < 5, além dos subgrupos das causas evitáveis, referem-se à organização das causas básicas entre os grupos e subgrupos^(1,9)^. No estudo, a partir do agregado temporal de 2010 a 2021, realizou-se o cálculo da proporção de óbitos fetais por grupo e subgrupo de evitabilidade da LBE-OF e da LBE < 5.

O teste de McNemar para amostras pareadas (antes e depois) foi aplicado para avaliar diferenças entre as categorias de evitabilidade das duas listas utilizadas. Considerou-se o antes, os grupos e subgrupos da LBE < 5, depois, a LBE-OF, admitindo-se nível de significância de 5% para todas as análises. Os dados foram analisados por meio do *Minitab Statistical Software*, versão 21.4.0.

Para a análise da tendência temporal, foram calculadas as taxas de mortalidade fetal total, precoce, tardia, evitável e mal definida, segundo as duas classificações e por ano de ocorrência. Para o cálculo, considerou-se o número de óbitos fetais dividido pelo total de nascimentos multiplicado por 1.000.

As tendências temporais da mortalidade fetal foram analisadas a partir do modelo de regressão *Joinpoint*, programa *Joinpoint,* versão 4.9.1.0, o qual testa se uma linha multissegmentada é estatisticamente melhor para descrever a evolução temporal do evento analisado do que uma linha reta ou com menos segmentos^(1)^. O modelo verifica se mudanças na tendência são estatisticamente significantes, e permite classificá-las em crescente, decrescente ou estacionária, a partir do teste de significância e da variação percentual anual (APC:annual percent change)^(1,15)^.

O programa também informa a variação percentual anual média do período analisado (AAPC: *average annual percent change*), que se refere à medida resumida da tendência, calculada a partir da média ponderada das APC^(1)^. A AAPC permite comparar tendências não constantes de diferentes grupos ao longo do período de interesse^(1)^. Na análise, as taxas de mortalidade fetal corresponderam às variáveis dependentes e os anos correspondentes, às variáveis independentes. Foi admitido o nível de significância estatística p < 0,05.

### Aspectos Éticos

A pesquisa foi desenvolvida a partir de dados secundários, está em conformidade com a Resolução 466/2012 do Conselho Nacional de Saúde, e obteve aprovação do Comitê de Ética em Pesquisa da Universidade Federal de Pernambuco no ano de 2022, sob o Parecer 6.317.405.

## RESULTADOS

No período estudado, ocorreram 2.490 óbitos fetais e 262.300 nascidos vivos, somando 264.790 NT de mães residentes no Recife. A TMF inicial (2010) foi de 8,6 e a final (2021), de 9,3 por 1.000 NT (Figura 1). Do total de óbitos, 1.874 (75,3%) corresponderam às tardias. Houve tendência temporal estacionária para a mortalidade fetal e seus componentes precoce e tardio (Tabela 1).

**Figura 1:** Tendência temporal da mortalidade fetal. Recife, Pernambuco, 2010–2021.

APC: variação percentual anual; Fonte: Sistema de Informações sobre Mortalidade (SIM) e Sistema de Informações sobre Nascidos Vivos (Sinasc).

**Tabela 1:** Tendência temporal da mortalidade fetal e de seus componentes precoce e tardia. Recife, Pernambuco, Brasil, 2010–2021.

Taxas de mortalidade (por 1.000 nascimentos totais)	TMF		Estimativas			*p-valor	Tendência
	2010	2021	APC	IC 95%			
Fetal total	8,6	9,3	–0,8	–2,0	0,4	**0,163**	**Estacionária**
Fetal precoce	2,1	2,3	–1,2	–3,9	1,5	**0,336**	**Estacionária**
Fetal tardia	6,1	7,0	–0,2	–1,8	1,4	**0,758**	**Estacionária**

APC: variação percentual anual; IC95%: intervalo de 95% de confiança;

*Modelo de Regressão *Joinpoint*; TMF: taxa de mortalidade fetal por mil nascimentos totais.

Fonte: Sistema de Informações sobre Mortalidade (SIM) e Sistema de Informações sobre Nascidos Vivos (Sinasc).

Segundo a LBE-OF, ocorreram 1.814 óbitos fetais evitáveis (72,9%), 471 por causas mal definidas (18,9%) e 205 (8,2%) por causas não claramente evitáveis. Com a LBE < 5, foram classificados 2.091 (84,0%) óbitos fetais evitáveis, 205 (8,2%) por causas mal definidas e 194 (7,8%) por causas não claramente evitáveis (Tabela 2).

**Tabela 2:** Evitabilidade dos óbitos fetais (n = 2.490) segundo a Lista Brasileira de Causas de Mortes Evitáveis para óbitos fetais (LBE-OF) e a Lista Brasileira de Causas de Mortes Evitáveis para menores de 5 anos (LBE < 5). Recife, Pernambuco, Brasil, 2010–2021.

Classificação de evitabilidade de causas de morte	LBE < 5		LBE-OF		p-valor*
	n	%	n	%	
1. Causas evitáveis	2.091	84,0	1.814	72,9	< 0,001
1.1 Reduzíveis por ações de imunoprevenção	0	–	0	–	
1.2. Reduzíveis por adequada atenção à mulher na gestação, no parto e ao recém-nascido					
1.2.1. Reduzíveis por adequada atenção à mulher na gestação	1.355	64,8	1.447	79,8	< 0,001
1.2.2. Reduzíveis por adequada atenção à mulher no parto	595	28,5	367	20,2	< 0,001
1.2.3. Reduzíveis por adequada atenção ao feto e ao recém-nascido	140	6,7	NA	NA	
1.3. Reduzíveis por ações adequadas de diagnóstico e tratamento	0	–	NA	NA	
1.4. Reduzíveis por ações adequadas de promoção à saúde e atenção à saúde	1	0,0	NA	NA	
2. Causas de morte mal definidas	205	8,2	471	18,9	< 0,001
3. Demais causas (não claramente evitáveis)	194	7,8	205	8,2	0,001
Total	2.490	100	2.490	100	

NA: não se aplica;

*Teste de McNemar.

Fonte: Sistema de Informações sobre Mortalidade (SIM) e Sistema de Informações sobre Nascidos Vivos (Sinasc).

Entre os óbitos evitáveis, predominaram, em ambas as classificações, os reduzíveis por adequada atenção à mulher na gestação, com 1.447 (79,8%) segundo a LBE-OF e 1.355 (64,8%) segundo a LBE < 5. O subgrupo reduzíveis por adequada atenção à mulher no parto apresentou 367 (20,2%) óbitos quando utilizada a classificação LBE-OF, e 595 (28,5%) óbitos com a LBE < 5 (Tabela 2).

Para a LBE < 5, as causas reduzíveis por adequada atenção ao feto e ao recém-nascido responderam por 141 (6,7%) óbitos fetais, e as causas reduzíveis, por ações adequadas de promoção à saúde, vinculadas a ações adequadas de atenção à saúde por 1 (0,05%) óbito fetal (Tabela 2).

Considerando o período total do estudo, a mortalidade fetal evitável apresentou tendência temporal crescente segundo a LBE-OF (APC: 2,1; p = 0,018) e tendência temporal estacionária segundo a LBE < 5 anos (Tabela 3).

**Tabela 3:** Tendência temporal da mortalidade fetal por causas evitáveis e mal definidas segundo a Lista Brasileira de Causas de Mortes Evitáveis para óbitos fetais (LBE-OF) e a Lista Brasileira de Causas de Mortes Evitáveis para menores de 5 anos (LBE < 5). Recife, Pernambuco, Brasil, 2010–2021.

Taxas de mortalidade (por 1.000 nascimentos totais)	Períodos de tendências	TMF		Estimativas			**p–Valor	Estimativas			**p–valor	Tendência
		Início	Final	APC	IC 95%			AAPC	IC 95%			
**Lista Brasileira de Causas de Mortes Evitáveis para óbitos fetais (LBE-OF)**												
Fetal evitáveis	2010–2021*	5,6	7,4	2,1*	0,4	3,8	**0,018**					**Crescente**
*Fetal reduzíveis por adequada atenção à mulher na gestação*	2010–2016*	3,9	6,6	7,9*	1,7	14,5	**0,019**	2,7	–1,2	6,8	**0,171**	**Estacionária**
	2016–2021	6,6	5,8	–3,1	–10,1	4,4	**0,348**					
*Fetal reduzíveis por adequada atenção à mulher no parto*	2010–2016*	1,6	0,9	–11,2*	–20,6	–0,7	**0,041**	–0,1	–7,7	8,2	**0,984**	**Estacionária**
	2016–2021	0,9	1,6	15,1	–2,1	35,3	**0,079**					
Fetal mal definidas	2010–2021*	2,5	1,1	–12,3*	–16,9	–7,4	**< 0,001**					**Decrescente**
**Lista Brasileira de Causas de Mortes Evitáveis para menores de 5 anos (LBE < 5)**												
Fetal evitáveis	2010–2021	7,2	7,6	–0,3	–1,7	1,2	**0,687**					**Estacionária**
*Fetal reduzíveis por adequada atenção à mulher na gestação*	2010–2016*	3,7	6,2	7,5*	0,7	14,9	**0,035**	2,0	–2,3	6,5	**0,363**	**Estacionária**
	2016–2021	6,2	5,5	–4,2	–11,9	4,1	**0,261**					
*Fetal reduzíveis por adequada atenção à mulher no parto*	2010–2018*	3,1	1,6	–8,7*	–11,1	–6,3	**< 0,001**	–5,8*	–9,3	–2,1	**0,002**	**Decrescente**
	2018–2021	1,6	1,6	2,6	–12,1	19,7	**0,711**					
*Fetal reduzíveis por adequada atenção ao feto e ao recém-nascido*	2010–2021	0,4	0,5	5,2	–0,9	11,7	**0,088**					**Estacionária**
Fetal mal definidas	2010–2012	0,9	1,4	19,8	–44,3	158,0	**0,548**	0,2	–16,0	19,4	**0,985**	**Estacionária**
	2012–2019*	1,4	0,2	–19,5*	–31,5	–5,3	**0,021**					
	2019–2021	0,2	0,9	79,7	–32,5	378,8	**0,172**					

APC: variação percentual anual; AAPC: variação percentual anual média; IC95%: intervalo de 95% de confiança;

*p < 0,05;

**Modelo de Regressão Joinpoint; TMF: taxa de mortalidade fetal por mil nascimentos totais.

Fonte: Sistema de Informações sobre Mortalidade (SIM) e Sistema de Informações sobre Nascidos Vivos (Sinasc).

Para as duas classificações, a tendência temporal dos óbitos reduzíveis por adequada atenção à mulher na gestação foi crescente entre 2010 e 2016 (APC: 7,9; p = 0,019; APC: 7,5; p = 0,035) e estacionária entre 2016 e 2021. Para os óbitos reduzíveis por adequada atenção à mulher no parto, houve tendência temporal decrescente em períodos superpostos: segundo a LBE-OF, entre 2010 e 2016 (APC: –11,2; p = 0,041), e a LBE < 5 anos, entre 2010 e 2018 (APC: -8,7; p < 0,001). Esse período de declínio foi seguido de tendência temporal estacionária de acordo com as duas classificações. Considerando o período total, houve tendência estacionária segundo a LBE-OF, e decrescente para a LBE < 5 anos (AAPC: –5,8; p < 0,002). Quanto aos óbitos reduzíveis por adequada atenção ao feto e ao recém-nascido, LBE < 5 anos, a tendência foi estacionária durante todo o período analisado (Tabela 3).

Houve tendência temporal decrescente para a mortalidade fetal por causas mal definidas segundo a LBE-OF (APC: –12,3; p < 0,001). Para a LBE < 5 anos, três tendências temporais foram identificadas: estacionária, entre 2010 e 2012; decrescente, entre 2012 e 2019 (APC: –19,5; p = 0,021); e estacionária, entre 2019 e 2021. Considerando o período total da série, a tendência temporal foi estacionária para a mortalidade fetal por causas mal definidas segundo a LBE < 5 anos (Tabela 3).

## DISCUSSÃO

A mortalidade fetal no Recife mostrou tendência temporal estacionária entre 2010 e 2021, sem variações significativas, mesmo durante os anos da pandemia pela covid-19. A mortalidade fetal precoce e tardia apresentaram tendência estacionária, com predominância dos óbitos fetais tardios, os quais corresponderam a mais de dois terços das mortes fetais.

A constatação de que a TMF no Recife não apresentou declínio significativo por mais de uma década, embora seja inferior às taxas observadas em Pernambuco, na região Nordeste e no Brasil como um todo, revela a necessidade de melhorias na assistência pré-natal e intraparto^(1,4)^. À semelhança do Recife, outros estudos realizados no país encontraram tendência temporal estacionária da mortalidade fetal: no Brasil, entre 1996 e 2015; em todas as regiões do país, entre 2000 e 2015; e no município de São Paulo, de 2007 a 2017^(1,4)^.

Esses resultados contrastam com a diminuição na mortalidade neonatal e infantil observada em diferentes áreas do Brasil, incluindo o Recife^(18-20)^. As ações de saúde da mulher e da criança, principalmente na atenção primária à saúde com a ampliação da Estratégia Saúde da Família, têm contribuído para o decréscimo nas taxas de mortalidade infantil nas últimas duas décadas^(1)^.

A despeito da redução nas taxas de mortalidade neonatal e infantil sugerir avanços na saúde materna e infantil, ratifica-se a necessidade de investimentos adicionais para prevenção dos óbitos fetais, especialmente os tardios^(1)^. A tendência estacionária da mortalidade fetal encontrada neste estudo, reafirma essa necessidade. A invisibilidade do óbito fetal em relação ao óbito infantil ocorre não somente no Brasil, mas no cenário internacional, considerando que a mortalidade fetal não foi incluída na agenda dos Objetivos de Desenvolvimento do Milênio (ODM) e não está presente nos Objetivos de Desenvolvimento Sustentável (ODS)^(1)^

Destaca-se que a Rede Cegonha foi implantada no Brasil, em 2011, como uma estratégia para assegurar a melhoria do acesso, da cobertura e da qualidade do acompanhamento pré-natal e da assistência ao parto, ao puerpério e à criança^(1)^. Entretanto, a avaliação do grau de implantação das boas práticas na atenção ao parto e nascimento em maternidades da Rede Cegonha aponta que, apesar dos avanços alcançados, há um longo caminho para qualificar esses serviços de saúde^(1)^.

Em relação à pandemia pela covid-19, no Brasil e no mundo, poucos estudos de séries temporais foram realizados para analisar o impacto desse evento na tendência da mortalidade fetal^(1,22)^. O presente estudo observou tendência estacionária da mortalidade fetal no Recife durante os anos de 2020 e 2021, semelhante ao encontrado para o Alabama, nos Estados Unidos^(1)^. Em contraste, uma pesquisa realizada em Moçambique identificou tendência decrescente da mortalidade fetal no período pandêmico^(1)^.

Estudos conduzidos para investigar possíveis diferenças no número de óbitos fetais durante a pandemia pela covid-19, em comparação com o período pré-pandêmico, encontraram resultados discordantes^(22-25)^. Esses achados têm sido explicados por fatores associados aos riscos relacionados à covid-19 durante a gravidez, às condições socioeconômicas e de saúde da população e às restrições no acesso aos cuidados de saúde impostas nesse período^(1,24)^.

O predomínio dos óbitos fetais tardios evidenciado no Recife está em concordância com o que ocorre no Brasil e regiões^(1)^. Diferentemente, em países de alta renda, a maior parte dos óbitos fetais são precoces e relativamente mais difíceis de prevenir, com as anomalias congênitas entre as principais causas de morte^(1,4)^.

Ressalta-se que no ano de 2021, a TMF tardia no Recife (7,0 por 1.000 NT) foi superior à do município de São Paulo (5,0), e próxima à do município do Rio de Janeiro (6,8) e a do Brasil (6,7)^(1)^. A comparação com países desenvolvidos, a exemplo da Suécia, que em 2019 apresentava 2,5 óbitos tardios por 1.000 NT, evidencia disparidades na mortalidade fetal e o potencial de prevenção desses óbitos^(1)^.

No que se refere à evitabilidade dos óbitos fetais ocorridos no Recife entre 2010 e 2021, a maior parte deles foi considerada evitável segundo as duas classificações. Esse resultado está em concordância com estudos realizados no Brasil e em outros países de baixa e média renda, nos quais a maioria das condições relacionadas às mortes fetais são evitáveis por meio da qualidade dos cuidados pré-natais e intraparto^(2,5,10,20)^.

A utilização da LBE < 5 anos resultou em uma frequência 11,1% maior de óbitos evitáveis em relação à LBE-OF. Esse resultado poderia sugerir uma maior sensibilidade da LBE < 5 anos na identificação da evitabilidade dos óbitos fetais, no entanto, deve-se à inclusão de causas consideradas mal definidas nos subgrupos reduzíveis por adequada atenção à mulher no parto e reduzíveis por adequada atenção ao feto e ao recém-nascido.

Entre os subgrupos de evitabilidade, o reduzíveis por adequada atenção à mulher na gestação apresentou a maior frequência de óbitos fetais de acordo com as duas listas. Concomitantemente, estudos nacionais apontam para evitabilidade da maior parte dos óbitos fetais, principalmente por meio da adequada assistência à gestação, seguida da adequada assistência ao parto^(5,10,20)^.

A constatação de que a maior parte dos óbitos fetais poderiam ter sido prevenidos, principalmente por meio da adequada atenção à gestação, aponta para deficiências desses cuidados de saúde. No Brasil, a avaliação da adequação da atenção pré-natal evidenciou fragilidades no acesso, na cobertura e na qualidade desse serviço. Reafirmando a necessidade de ações governamentais que garantam uma assistência pré-natal de qualidade^(1)^.

A pesquisa que comparou os resultados da LBE < 5 anos e da LBE-OF na classificação de 2.585 óbitos fetais ocorridos em 2018 no estado do Rio de Janeiro encontrou para o grupo de causas mal definidas os percentuais de 6,7% e 35,5%, respectivamente^(10)^. Em concordância, no presente estudo, ao se utilizar a LBE-OF, o grupo de causas mal definidas apresentou maior percentual de óbitos em comparação a LBE < 5 anos.

Esses resultados são explicados pelas diferenças na alocação de algumas causas mal definidas. Classificações de evitabilidade condizentes com a fisiopatologia do óbito fetal são importantes para dimensionar o real potencial de prevenção dos óbitos, comunicar fragilidades na atribuição das causas, e indicar a necessidade de melhorias na disponibilidade e/ou qualidade dos exames *post mortem* ^(1,10)^.

No Brasil, 36,6% dos óbitos fetais ocorridos entre 2013 e 2016 tiveram como causa básica a hipóxia intrauterina não especificada (CID-10 P20.9) ou a morte fetal de causa não especificada (CID-10 P95)^(1)^. No município de São Paulo, um estudo que analisou as causas básicas de 720 óbitos fetais registrados entre 2012 e 2014 identificou essas mesmas como causas de 44,7% dos óbitos^(1)^. No município do Rio de Janeiro, as causas mal definidas corresponderam a 25,8% dos óbitos fetais registrados em 2018^(1)^. Considerando os resultados dessas pesquisas, as quais classificaram a hipóxia intrauterina não especificada (CID-10 P20.9) no grupo de causas mal definidas, sugere-se que no Recife há um menor percentual dessas causas, o que pode estar relacionado, entre outros fatores, à efetividade da vigilância do óbito^(1)^.

A análise das tendências temporais da mortalidade fetal evitável segundo a LBE < 5 anos e a LBE-OF revelou tendências distintas, crescente segundo a LBE-OF e estacionária conforme a LBE < 5 anos. É possível que o aumento evidenciado na mortalidade fetal evitável segundo a LBE-OF esteja relacionado à tendência decrescente das causas mal definidas. Assim, a partir da melhoria na atribuição das causas, um maior número de óbitos passou a ser classificado em evitável. Em contraste, para a LBE < 5 anos, ao considerar o período total da série, as tendências temporais da mortalidade fetal evitável e mal definida mantiveram-se estacionárias, sugerindo que essa lista não foi capaz de detectar todo o avanço alcançado na atribuição das causas básicas. No entanto, a tendência decrescente detectada na mortalidade fetal por causas mal definidas segundo a LBE < 5 anos, no período de 2012 a 2019, reafirma a importância da vigilância do óbito para a qualificação das causas de morte no município.

Considerando a mortalidade fetal reduzível por adequada atenção à gestação, no período total da série, constatou-se tendência estacionária segundo as duas classificações, o que ratifica a necessidade de investimentos que garantam, além da captação precoce e do número mínimo de seis consultas, a qualidade desses atendimentos^(1)^. À semelhança dos países desenvolvidos, os resultados apontaram maior taxa de mortalidade fetal relacionada à qualidade da atenção à gestação em detrimento da atenção ao parto^(1)^.

Ao considerar o período total do estudo, a tendência estacionária do componente adequada atenção ao parto segundo a LBE-OF ressalta a importância de melhorias contínuas nos cuidados intraparto. Em contraste, segundo a LBE < 5 anos, ao considerar o período total da série, houve tendência decrescente da mortalidade fetal reduzível por adequada atenção ao parto. Sugere-se que essa diferença esteja relacionada à diminuição na atribuição da causa hipóxia intrauterina não especificada (CID-10 P20.9), classificada na LBE < 5 anos como reduzível por adequada atenção ao parto.

No Brasil e no mundo, a hipóxia tem sido frequentemente relatada como causa dos óbitos fetais quando, na verdade, refere-se à via patológica pela qual a morte ocorre^(6,11,29)^. A hipóxia, assim como as demais causas mal definidas, não é útil para identificar os fatores determinantes do óbito e não contribui no planejamento de intervenções e políticas de saúde^(1,6)^.

Entender o que favorece a atribuição das causas mal definidas é o primeiro passo para intervir na sua redução. Isso pode estar relacionado à má qualidade de registros clínicos, à deficiente capacitação dos profissionais para identificar a causa básica e à indisponibilidade ou a baixa qualidade de exames *post mortem* ^(1,6)^. Em menor proporção, as causas mal definidas estão associadas aos óbitos fetais inexplicáveis, para os quais, mesmo após a realização de exames *post mortem* adequados, as causas não foram esclarecidas^(1,11)^.

Quanto às limitações deste estudo, ressalta-se a utilização de dados secundários, os quais podem conter incompletude nos registros e falhas na cobertura. Entretanto, a completude e concordância dos Sistemas de Informação sobre Mortalidade e Nascidos Vivos do Recife são considerados adequados, o que ratifica a sua aplicabilidade para aferição da situação de saúde da população^(1)^.

## CONCLUSÃO

A estagnação da tendência da mortalidade fetal por mais de uma década e a evitabilidade da maior parte dos óbitos fetais, principalmente por meio da adequada atenção ao pré-natal e ao parto, revelam a necessidade de qualificar a assistência pré-natal e de investimentos contínuos nos cuidados intraparto. Profissionais capacitados e em número adequado, o acesso aos exames pré-natais em tempo oportuno, a classificação do risco gestacional, o manejo apropriado das gestações de alto risco, instalações e recursos materiais para oferecer cuidados de qualidade são indispensáveis para alcançar a redução dos óbitos fetais.

As diferenças encontradas na classificação de evitabilidade dos óbitos fetais segundo a LBE < 5 anos e a LBE-OF, principalmente a maior frequência de óbitos classificados no grupo de causas mal definidas ao se utilizar a LBE-OF, evidenciam a potencialidade dessa classificação na monitorização da qualidade da informação sobre as causas dos óbitos fetais. Assim, a utilização da LBE-OF na vigilância do óbito fetal pode contribuir para o planejamento de ações que favoreçam a correta identificação das causas básicas de morte e, portanto, para a redução das causas mal definidas.

A tendência temporal decrescente das causas mal definida, evidenciada ao utilizar a LBE-OF, revela os avanços alcançados na atribuição das causas básicas dos óbitos fetais no Recife, o que sugere a efetividade da vigilância do óbito fetal e reforça a importância desse serviço e de investimentos que propiciem o seu fortalecimento. Esse resultado ratifica a possibilidade de outras cidades também reduzirem as causas mal definidas.
